# An Eight-Membered Ring Molecular Framework Based on Carbazole for the Development of Electroluminescent Materials

**DOI:** 10.3390/molecules30030716

**Published:** 2025-02-05

**Authors:** An Yan, Shipan Xu, Xuyang Du, Chengyun Zhu, Shengli Li, Xiaolong Yang, Guijiang Zhou, Yuanhui Sun

**Affiliations:** School of Chemistry, Xi’an Key Laboratory of Sustainable Energy Material Chemistry, Engineering Research Center of Energy Storage Materials and Devices, Ministry of Education, Xi’an Jiaotong University, Xi’an 710049, China

**Keywords:** organic light-emitting diode, eight-membered ring, rigid structure, nonplanar structure, device efficiency

## Abstract

The organic light-emitting diode (OLED) has been regarded as the most prominent product in the current market of organic electronics, which has attracted growing attention because of their applications in full-color displays and solid-state lighting. Organic materials that exhibit strong luminescence in the solid state constitute the core position of OLED. Extensive research efforts to probe the structure of organic luminescent materials have attracted considerable attention to the conjugated fusion ring architecture. This is because it can confer molecular rigidity and helps to inhibit intermolecular interactions and non-radiative transitions, thus enhancing the performance of luminescent materials. Here, we use an efficient and simple method to construct an eight-membered ring molecular framework based on carbazole. Moreover, we have introduced groups with different electron-withdrawing abilities to develop a series of luminescent molecules. The results show that the nonplanar structure based on the eight-membered ring suppresses fluorescence quenching caused by molecular aggregation. As the doping concentration increases, the electroluminescence spectrum remains basically unchanged, indicating that the eight-membered ring structure can effectively suppress the intermolecular interaction. Notably, DCBz-pm exhibits deep blue emission with a Commission Internationale de l’Eclairage (CIE) coordinate of (0.158, 0.046), which nearly meets the BT. 2020 standards. The DCBz-CN device reaches a maximum external quantum efficiency (EQE) of 4.36%. These results offer a new design strategy for improving the performance of OLEDs.

## 1. Introduction

**Scheme 1:**
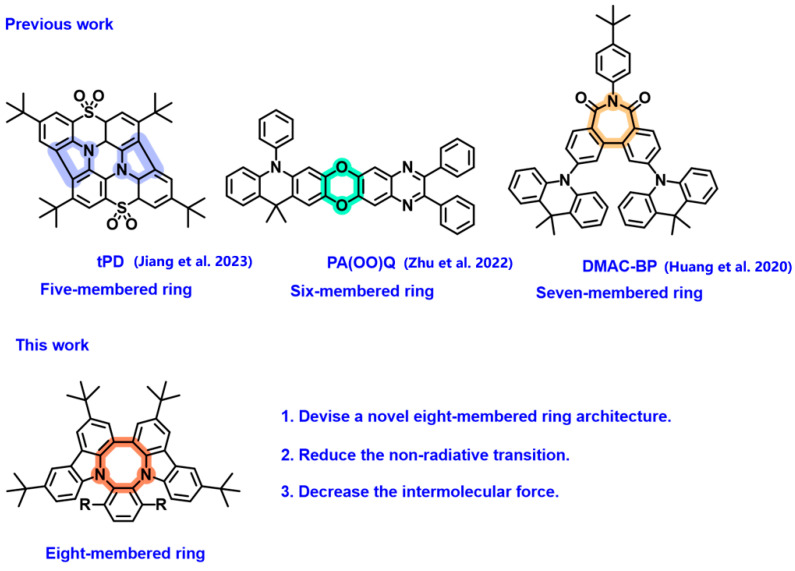
Represented structures of five-, six-, and seven-numbered rings in previous work and the eight-membered ring molecular frame based on the carbazole group developed in this work [21,22,23].

Organic light-emitting diodes (OLEDs) stand out as the most promising contenders for the next-generation display and illumination technologies thanks to their high efficiency, flexible device architectures, and diverse multicolor emissions [1,2,3]. OLEDs are expected to become the future direction of development [4,5,6]. Moreover, OLEDs will attract increasing attention from industry stakeholders and the scientific research community [7,8,9,10]. The emissive layer is the most prominent layer of OLEDs, and the device emission color, contrast ratio, and external efficiency are dependent on the luminescent materials of this layer [11]. However, in the doping process, the aggregation quenching effect caused by the intermolecular interaction force makes emitting materials challenging [12,13,14]. Generally, flexible molecular skeletons suffer from severe structural relaxation, leading to reduced performance in OLED. Therefore, it is wise to develop novel chemical structures in place of a flexible molecular skeleton. Extensive research on the structure of organic luminescent materials has drawn significant attention to the conjugated fused-ring structure. Up to now, the construction of nonplanar rigid structures in molecules has become a good strategy to solve the problem [15,16,17], as it contributes to suppressing intermolecular interactions and non-radiative transitions, thereby enhancing the photoluminescence quantum yield (PLQY) and maximum EQE [18,19,20]. In 2023, Jiang et al. synthesized a five-membered ring rigid structure (tPD) (Scheme 1), which effectively limited the relaxation vibrations of the excited state and minimized non-radiative transitions, significantly improving the EQE to 22% and mitigating the efficiency roll-off [21]. This demonstrates the effectiveness of rigid structures in enhancing the performance of OLEDs. In 2022, a six-membered ring rigid skeleton structure was formed by partially connecting the donor and acceptor using two oxygen atoms (PA(OO)Q), achieving the maximum EQE of 19.4% [22]. This shows that specific molecular designs can lead to significant improvements in OLED performance. In 2020, You et al. developed a twisted heptagonal diimide acceptor with well-balanced structural rigidity and rotatability for the synthesis of a highly efficient aggregation-induced delayed fluorescence material (DMAC–BPI) [23]. The rigid structure can inhibit the rotation of intramolecular groups and reduce the rate of nonradiative transitions. Additionally, the moderate flexibility can inhibit the strong π-π packing between molecules and reduce the exciton quenching in the aggregate state, which greatly improve the performance of OLEDs [24,25,26].

Currently, among the reported rigid structure molecules, to the best of our knowledge, no research has incorporated the rigid structure of the eight-membered ring into the study of OLEDs. In this work, we added two di-*tert*-butylcarbazole units to the ortho positions of the benzene core and locked the carbazole units through a Scholl cyclization reaction to construct a rigid eight-membered ring structure. Different groups were linked by the Suzuki reaction to obtain compounds with various luminescence colors. The nonplanar structure of the eight-membered ring effectively suppresses the close π-π stacking interactions between molecules, reduces intense molecular vibrations, and minimizes non-radiative transitions. Simultaneously, it also limits the excessive rotation of the structural elements in the molecule and inhibits the aggregation quenching of the molecule. Surprisingly, DCBz-pm exhibits deep blue emission with a CIE coordinate of (0.158, 0.046), which meets the BT. 2020 standards [5]. The DCBz-CN device reaches a maximum EQE of 4.36%, which is close to the theoretical limit of EQE (∼5%) for fluorescent OLEDs without any special light extraction technique. Therefore, this work offers a new approach to developing potentially high-performance organic emitters for OLED applications.

## 2. Results and Discussion

### 2.1. Synthesis and Characterization

**Scheme 2:**
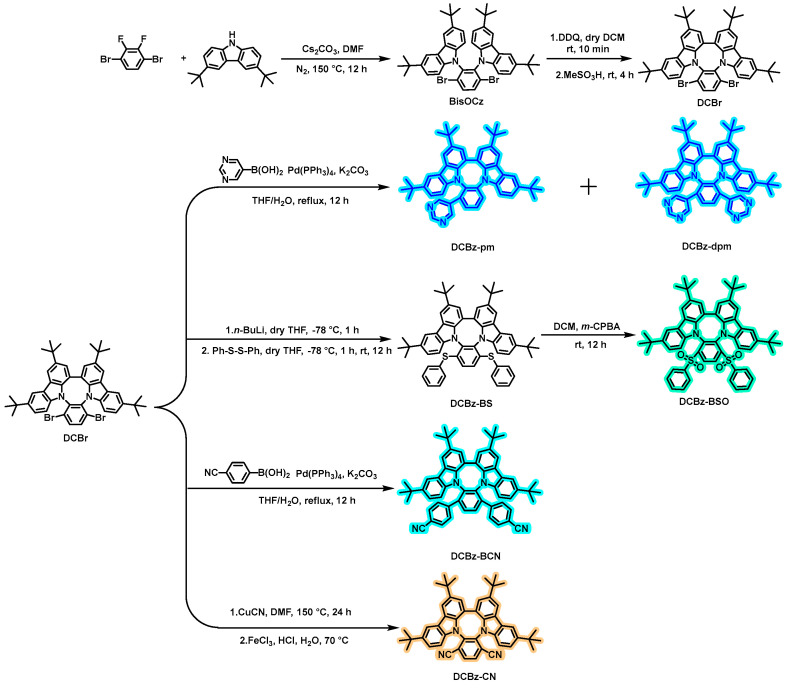
Synthetic route of the emitters based on the eight-membered ring.

The synthetic route is shown in Scheme 2. Detailed information is provided in Supplementary Information (SI). The chemical structures of the emitters based on the eight-membered ring were characterized by nuclear magnetic resonance (NMR) and mass spectrometry (MS).

### 2.2. Thermal and Photophysical Properties

**Figure 1:**
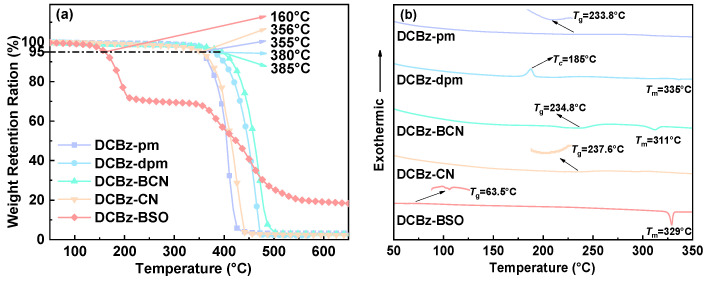
(**a**) TGA and (**b**) DSC curves of the eight-membered ring group.

The thermal properties of the five materials are estimated by thermalgravimetric analysis (TGA) and differential scanning calorimetry (DSC). According to thermogravimetric analysis (TGA) results, the decomposition temperatures (*T*_d_) of DCBz-pm, DCBz-dpm, DCBz-BCN, and DCBz-CN at a 5% weight loss are 355, 380, 385, and 356 °C, respectively (Figure 1a). The weight percentage above 400 °C is nearly zero, which is attributed to the sublimation of the material rather than decomposition. This indicates their good thermal stability. In contrast, the *T*_d_ of DCBz-BSO measured by TGA at 5% weight loss is 160 °C, indicating poor thermal stability. Weight percentage analysis shows that the decomposition is ascribed to the destruction of the sulfone unit. From the DSC curves (Figure 1b), except for DCBz-dpm, the glass transition temperatures (*T*_g_) of other luminescent materials can be observed. Specifically, DCBz-pm, DCBz-BCN, DCBz-CN, and DCBz-BSO show different *T*_g_ values at 233.8, 234.8, 237.6, and 63.5 °C, respectively. Among them, the *T*_g_ temperature of DCBz-BSO is relatively low, indicating poor thermal stability. Additionally, the crystallization temperature (*T*_c_) of DCBz-dpm is 185 °C. No obvious crystallization temperature was observed for other materials. The melting temperatures (*T*_m_) of DCBz-dpm, DCBz-BCN, and DCBz-BSO are 335, 311, and 329 °C, respectively. In conclusion, these results suggest that DCBz-pm, DCBz-dpm, DCBz-BCN, and DCBz-CN all have good thermal stability.

**Figure 2:**
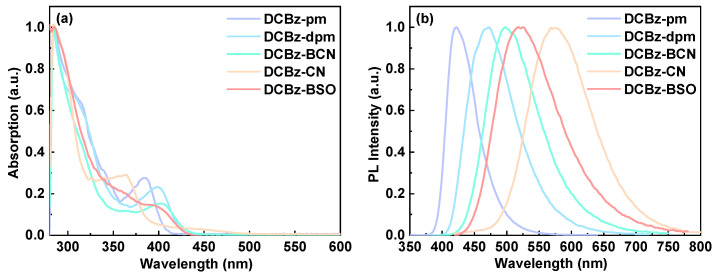
The (**a**) UV–vis absorption and (**b**) PL spectra in toluene solution.

The ultraviolet–visible (UV–vis) absorption and photoluminescence (PL) spectra of DCBz-pm, DCBz-dpm, DCBz-BCN, DCBz-CN, and DCBz-BSO were measured in toluene solution. The photophysical data are summarized in Table 1. The highly intense absorption peaks below 320 nm are attributed to the delocalized π–π* transition originating from the eight-membered ring. Additionally, the weak intensity absorption peaks ranging from 360 nm to 400 nm are ascribed to the charge-transfer (CT) transition from the electron-rich eight-membered ring to the electron-deficient groups like pyrimidine, cyano, and sulfone (Figure 2a). In the PL spectra, compared to DCBz-pm, DCBz-dpm exhibits a significant redshift and broadening as the number of electron-missing bases increases (Figure 2b). When compared the emitters of DCBz-dpm, DCBz-BCN, DCBz-CN, and DCBz-BSO, they showed significant redshift and broadening as the electron deficiency intensifies. The observed redshift and spectral broadening arise from the strong CT state between the electron-rich eight-membered ring and the electron-deficient moiety [27,28]. The transient photoluminescence decay curves of the five luminescent materials demonstrated that they all had very short lifetimes of only several nanoseconds under degassed conditions in toluene solution (Figure S1). No delayed long lifetimes were observed, which suggested that these emitters were fluorescent materials.

### 2.3. Theoretical Calculation

**Figure 3:**
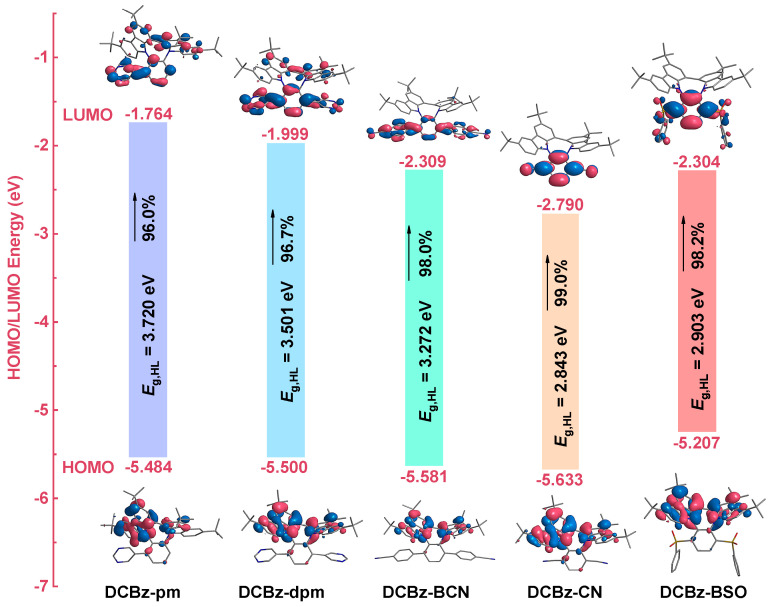
Optimized S_0_ geometries, energy levels, and frontier molecular orbital distributions.

**Figure 4:**
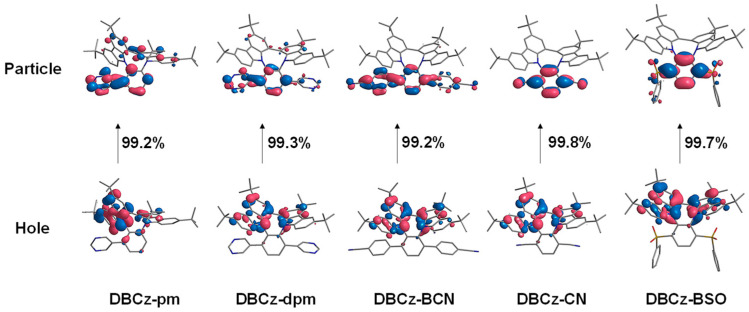
The NTO distribution of S_0_→S_1_ based optimized the S_1_ configuration.

To investigate the geometrical and electronic properties of the five compounds, a theoretical calculation was performed via density functional theory (DFT) and time-dependent DFT (TD-DFT). Non-metal atoms of C, H, N, O, and S were calculated based on B3LYP/6-31G(d, p). The excitation behaviors of the compounds were calculated by the TD-DFT method based on optimized geometries at the ground states. All calculations were carried out by using the Gaussian 09 program. The optimized ground-state (S_0_) structures are shown in Figure 3. The nonplanar structure of the rigid eight-membered ring is conducive to the inhibition of fluorescence quenching induced by molecular aggregation. The highest occupied molecular orbital (HOMO) is located mainly on the eight-membered ring rigid skeleton with slight scattering on the pyrimidine ring, while the lowest unoccupied molecular orbital (LUMO) is concentrated on the electron-deficient unit. As the degree of electron deficiency increases, the LUMO energy level decreases and the separation between HOMO and LUMO tends to be complete. Furthermore, the theoretical calculations show that the HOMO→LUMO transition of these compounds exerts a dominant influence on the S_0_→S_1_ transition, and the contribution rates are 96.0%, 96.7%, 98.0%, 99.0%, and 98.2%, respectively. Therefore, all compounds exist in the CT state [29]. The calculations of the natural transition orbital (NTO) from S_0_ to S_1_ based on the optimized S_1_ configuration are shown in Figure 4. The proportions from hole to particle are 99.2%, 99.3%, 99.2%, 99.8%, and 99.7%, respectively. The hole and electron are almost completely separated, which is similar to the frontier molecular orbital (FMO) distribution of the ground state, and thus all of these emitters show CT state [30,31].

### 2.4. Electrochemical Properties

**Figure 5:**
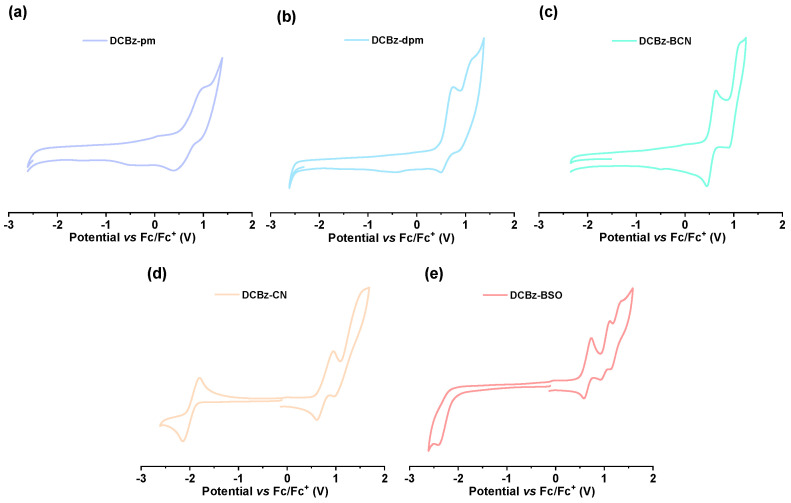
CV curves of (**a**) DCBz-pm, (**b**) DCBz-dpm, (**c**) DCBz-BCN, (**d**) DCBz-CN, and (**e**) DCBz-BSOrelative to ferrocene.

Electrochemical properties were characterized by cyclic voltammetry (CV). As shown in Figure 5, the relevant data are listed in Table 2. Oxidation signals near 0.7 V are observed for the five compounds, which are attributed to the oxidation of the eight-membered ring unit with a HOMO level of about −5.5 eV. A reversible reduction signal was observed in DCBz-CN (Figure 5d), while an irreversible reduction signal was observed in DCBz-BSO (Figure 5e). The energy gap differences were calculated to be 2.75 and 3.08 eV, respectively, based on the measured electrochemical properties. However, no significant reduction signals of DCBz-pm (Figure 5a), DCBz-dpm (Figure 5b), and DCBz-BCN (Figure 5c) were observed. They are attributed to pyrimidine’s weak electron deficiency ability, which is difficult to reduce. At the same time, the electrons of the two benzene rings in DCBz-BCN are delocalized to the cyanogen unit, resulting in an increase in electron cloud density of the two cyanogen units. This makes it difficult for the compound to be reduced. Therefore, the energy gap differences calculated from the starting bands of their UV–vis absorption spectra for DCBz-pm, DCBz-dpm, and DCBz-BCN are 3.07, 2.94, and 2.90 eV, respectively.

### 2.5. Electroluminescent Properties

**Figure 6:**
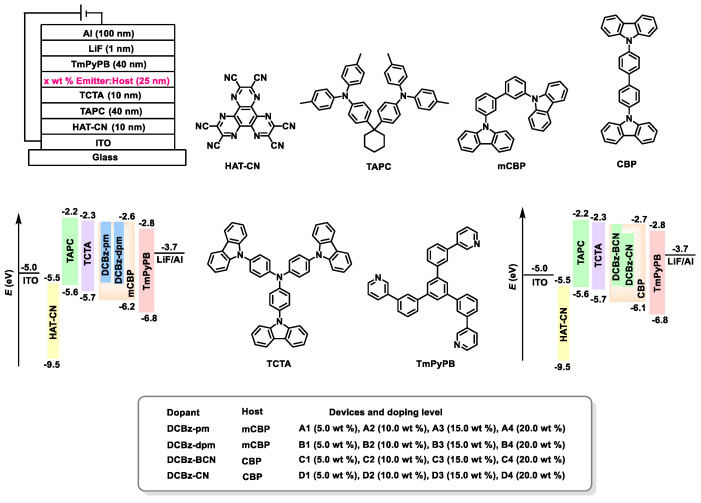
Device structures and functional materials diagram.

DCBz-pm, DCBz-dpm, DCBz-BCN, and DCBz-CN exhibit good thermal stability and are utilized for fabricating devices using a vacuum deposition process. The device structures and the molecular structures of these functional materials are illustrated in Figure 6. The optimized device structure is ITO (indium tin oxide)/HAT-CN (1,4,5,8,9,11-Hexaazatriphenylenehexacarbonitrile) (10 nm)/TAPC (bis [4-(N,N-ditolylamino)-phenyl]cyclohexane) (40 nm)/TCTA (tris[4-(9H-carbazol9-yl)phenyl]amine) (10 nm)/Emitter (5.0/10.0/15.0/20.0 wt%): Host (25 nm)/TmPyPB (1,3,5-tri(m-pyrid-3-yl-phenyl)benzene) (40 nm)/LiF (1 nm)/Al (100 nm). Compound HAT-CN was used as a hole injection layer (HIL) material because of its suitable energy level that matches well with the anode and the adjacent organic layers to easily accept holes from the anode and then transfer them to the next organic layer with a relatively small energy barrier. Compound TAPC was used as hole-transporting material for its relatively high hole mobility. Compound TCTA also has relatively high hole mobility and its suitable HOMO energy level between TAPC and the EML could help the hole injection into the EML. Compound TmPyPB was used as electron-transporting as well as a hole blocking layer because of its relatively high electron mobility and its deep HOMO energy level. Compound LiF was served as electron-injection material since it is well-aligned LUMO with the adjacent organic semiconductor layers and the cathode which is favorable for electron injection. As for the host material, compared with CBP (4,4′-Bis (N-carbazolyl)-1,1′-biphenyl), the host material mCBP (3,3′-Di (9H-carbazol-9-yl)-1,1′-biphenyl) has a higher triplet exciton energy. Therefore, for the devices based on blue emitter (DCBz-pm and DCBz-dpm), mCBP is used as the host material [32,33], and they are named as A1–A4 and B1–B4. For the devices based on green emitter (DCBz-BCN and DCBz-CN), CBP is used as the host material [34,35], and they are named as C1–C4 and D1–D4.

**Figure 7:**
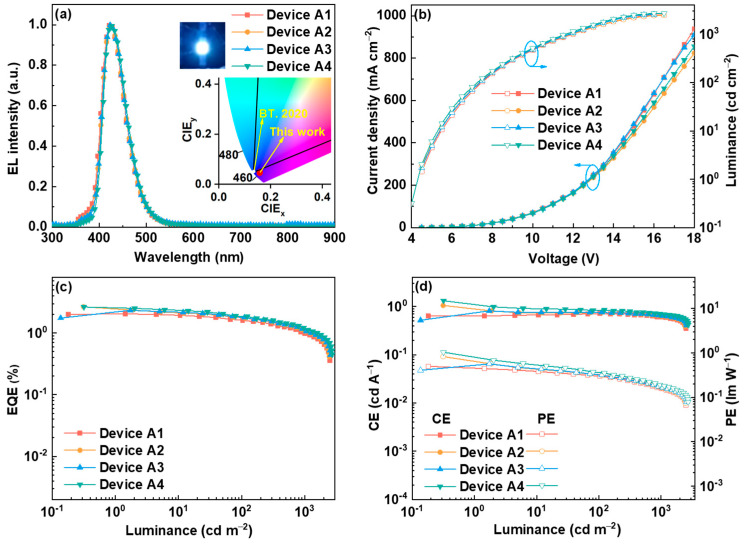
EL performance of DCBz-pm: (**a**) EL spectra, (**b**) relationship of current density (*J*)-voltage (*V*)-luminance (*L*), (**c**) relationship of EQE vs. luminance, and (**d**) relationship of CE and PE vs. luminance.

**Figure 8:**
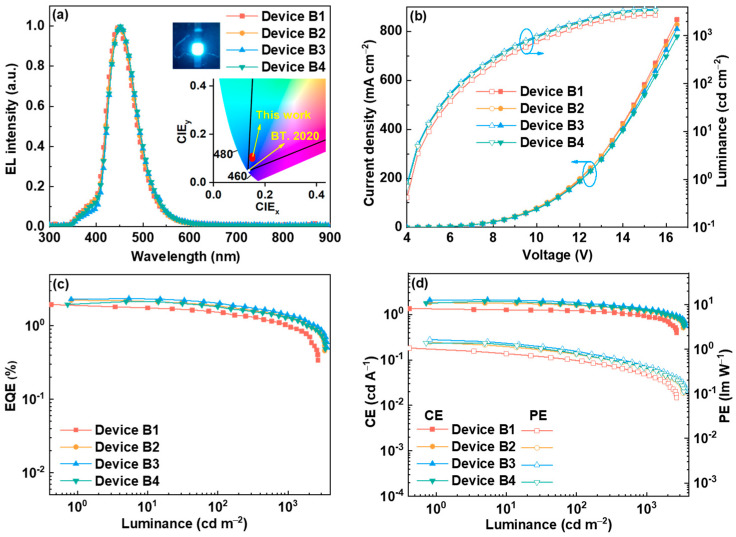
EL performance of DCBz-dpm: (**a**) EL spectra, (**b**) relationship of current density (*J*)-voltage (*V*)-luminance (*L*), (**c**) relationship of EQE vs. luminance, and (**d**) relationship of CE and PE vs. luminance.

In the electroluminescence (EL) spectra, the devices based on DCBz-pm exhibit a deep blue emission at approximately 425 nm (Figure 7). When the doping concentration is 20%, the maximum EQE is 2.52%. The CIE color coordinate (0.158, 0.046) closely approximates the blue light coordinates (0.131, 0.046) as specified by the BT. 2020 standard. In the case of the devices based on DCBz-dpm, the maximum emission wavelength is approximately 450 nm (Figure 8). When the doping concentration is 15%, the maximum EQE is 2.35%. Even when the doping concentration is increased to 20%, there is no significant decrease. The corresponding data are listed in Table 3. The results show that despite the increase in doping concentration, the EL spectrum remains basically unchanged, which indicates that the rigid nonplanar eight-membered ring skeleton effectively inhibits intermolecular interactions and reduces the molecular aggregation quenching effect.

**Figure 9:**
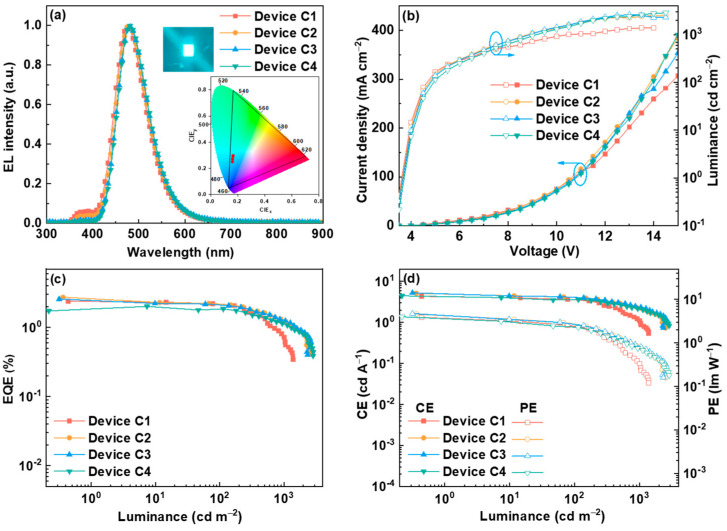
EL performance of DCBz-BCN: (**a**) EL spectra, (**b**) relationship of current density (*J*)-voltage (*V*)-luminance (*L*), (**c**) relationship of EQE vs. luminance, and (**d**) relationship of CE and PE vs. luminance.

**Figure 10:**
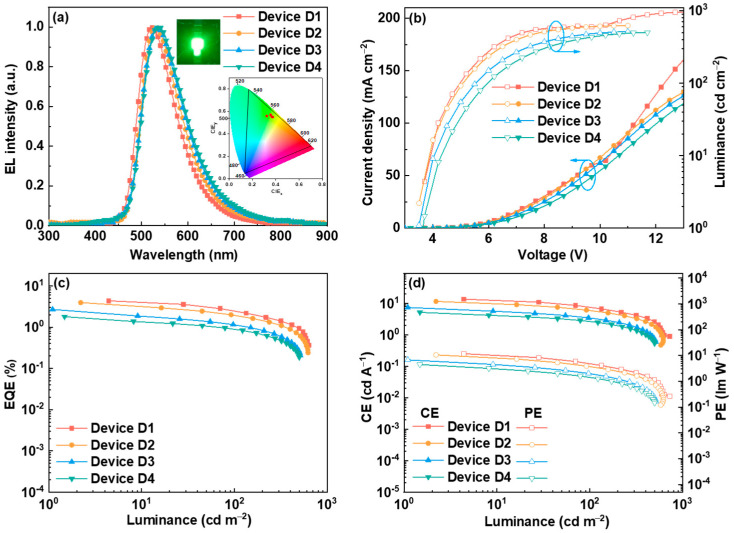
EL performance of DCBz-CN: (**a**) EL spectra, (**b**) relationship of current density (*J*)-voltage (*V*)-luminance (*L*), (**c**) relationship of EQE vs. luminance, and (**d**) relationship of CE and PE vs. luminance.

The emission peaks of the devices based on DCBz-BCN and DCBz-CN are situated at approximately 480 nm and 520 nm. The corresponding data are listed in Table 4. The peak at 390 nm is attributed to the luminescence of the host material CBP at low doping concentrations (Figure 9). With the increase in doping concentration, the EL spectrum of DCBz-CN shows a more pronounced redshift than DCBz-BCN (Figure 10). This phenomenon can be attributed to the torsion angle between the phenyl cyan unit and the central core of the eight-membered ring in the DCBz-BCN, which creates steric hindrance and prevents the molecules from packing closely [36,37]. On the contrary, due to the smaller cyano unit in the DCBz-CN molecule, it does not provide a significant hindrance to molecular stacking. Compared to their PL spectra, the EL spectrum of DCBz-BCN and DCBz-CN exhibits a substantial blue shift. This transition is attributed to the relatively low emission wavelength in the solution being related to the geometric changes in the excited state. After being doped into a solid film, the non-radiative decay caused by structural distortion can be greatly suppressed [38,39]. At the doping concentration of 10.0 wt%, the DCBz-BCN device demonstrated optimal performance, with maximum EQE reaching 2.74%. The doping concentration of DCBz-CN-based device D1 was 5.0 wt%, and the maximum EQE of device D1 reached 4.36%, which is near to the theoretical limit of EQE (∼5%) for fluorescent OLEDs without any special light extraction technique.

## 3. Conclusions

In this work, a nonplanar rigid eight-membered ring structure is reported, in which synthesized emitters exhibit excellent photophysical and electrochemical properties. The devices based on DCBz-pm exhibit deep blue emission at approximately 425 nm. The CIE color coordinate (0.158, 0.046) closely approximates the blue light coordinates (0.131, 0.046) as specified by the BT.2020 standard. The DCBz-CN devices exhibit green emission at 530 nm and have a maximum EQE of 4.36% at a doping concentration of 5.0 wt%. Despite the increase in doping concentration, the respective EL spectra remain basically unchanged, indicating that the rigid eight-membered ring structure can effectively inhibit intermolecular interactions. We firmly believe that the design concept of the nonplanar rigid eight-membered ring structure will greatly facilitate the development of electroluminescent materials for display applications in the future.

## Figures and Tables

**Table 1:** Photophysical and thermal data.

Compound	*λ*_abs_ (nm) ^a^	λ_em_ (nm) ^a^	τ (ns) ^a^	PLQY ^b^	*T*_d_ (°C)
DCBz-pm	285(4.36), 313(4.16), 385(3.78)	418	4.59	0.45	355
DCBz-dpm	284(4.43), 308(4.38), 398(3.91)	468	12.30	0.49	380
DCBz-BCN	285(4.68), 315(4.34), 402(3.88)	497	14.37	0.48	385
DCBz-CN	285(4.41), 364(3.89)	573	7.47	0.69	356
DCBz-BSO	285(4.42), 397(3.57)	520	15.00	0.36	160

^a^ Measured in toluene at room temperature, the logarithm of the molar extinction coefficient (log*ε*) is represented in parentheses. ^b^ Absolute photoluminescence quantum yield measured in toluene.

**Table 2:** Redox properties of these luminescent materials.

Compound	*E*_ox_ ^a^ (V)	*E*_red_ ^a^ (V)	*E*_HOMO_ ^b^ (eV)	*E*_LUMO_ (eV)	*E*_g(CV)_ (eV)
DCBz-pm	0.75	/	−5.44	−2.37 ^d^	3.07 ^c^
DCBz-dpm	0.81	/	−5.40	−2.46 ^d^	2.94 ^c^
DCBz-BCN	0.65	/	−5.31	−2.41 ^d^	2.90 ^c^
DCBz-CN	0.89	−1.86	−5.58	−2.83 ^e^	2.75 ^f^
DCBz-BSO	0.77	−2.31	−5.46	−2.38 ^e^	3.08 ^f^

^a^ Relative to ferrocene. ^b^ *E*_HOMO_ = −(4.8 + *E*_ox_) eV. ^c^ *E*_g(abs)_ calculated from the onset of the UV–vis absorption spectra. ^d^ *E*_LUMO_ = *E*_HOMO_ + *E*_g(CV)_. ^e^ *E*_LUMO_ = −(*E*_red_ + 4.8) eV. ^f^ *E*_g(CV)_ = E_LUMO_ − *E*_HOMO_.

**Table 3:** EL performance of devices A1–A4 and B1–B4.

Device (Doping Level)	*V*_on_ (V)	*L* (cd m^−2^)	EQE (%)	CE (cd A^−1^)	PE (lm W^−1^)	*λ*_EL_ (nm)	CIE (*x*, *y*)
A1 (5.0 wt%)	4.5	2508	2.05	0.71	0.44	423	(0.158, 0.046)
A2 (10.0 wt%)	4.5	2446	2.35	0.81	0.57	424	(0.160, 0.046)
A3 (15.0 wt%)	4.5	2557	2.35	0.81	0.57	425	(0.158, 0.046)
A4 (20.0 wt%)	4.5	2720	2.52	0.98	0.70	426	(0.157, 0.047)
B1 (5.0 wt%)	4.5	2679	1.80	1.28	1.06	446	(0.153, 0.100)
B2 (10.0 wt%)	4.0	3330	2.23	1.82	1.43	450	(0.151, 0.103)
B3 (15.0 wt%)	4.0	3576	2.35	2.10	1.64	453	(0.150, 0.108)
B4 (20.0 wt%)	4.0	3400	2.13	1.95	1.38	454	(0.152, 0.115)

**Table 4:** EL performance of devices C1–C4 and D1–D4.

Device (Doping Level)	*V*_on_ (V)	*L* (cd m^−2^)	EQE (%)	CE (cd A^−1^)	PE (lm W^−1^)	*λ*_EL_ (nm)	CIE (*x*, *y*)
C1 (5.0 wt%)	3.5	1387	2.31	4.00	3.10	472	(0.163, 0.250)
C2 (10.0 wt%)	3.5	2530	2.74	5.14	4.62	479	(0.164, 0.271)
C3 (15.0 wt%)	3.5	2543	2.61	5.03	4.52	480	(0.166, 0.285)
C4 (20.0 wt%)	3.5	2549	2.01	4.44	4.00	480	(0.168, 0.296)
D1 (5.0 wt%)	3.7	951	4.36	13.83	11.74	520	(0.323, 0.558)
D2 (10.0 wt%)	3.5	621	3.96	11.75	10.55	530	(0.355, 0.566)
D3 (15.0 wt%)	3.5	513	2.69	7.52	6.76	533	(0.367, 0.561)
D4 (20.0 wt%)	3.7	500	1.79	5.26	4.47	534	(0.374, 0.561)

## Data Availability

The data presented in this study are available in Supplementary Materials.

## Supplementary Materials

The following supporting information can be downloaded at: https://www.mdpi.com/article/10.3390/molecules30030716/s1, General experimental information, Figure S1: PL decay curves of these five compounds. Figures S2–S16: NMR and MS spectra of these five compounds.
