# Supplementary material for: An Eight-Membered Ring Molecular Framework Based on Carbazole for the Development of Electroluminescent Materials
Source: Molecules. 2025 Feb 5;30(3):716. doi: 10.3390/molecules30030716 (PMC11819814; doi:10.3390/molecules30030716)
Supplement: Supplementary file 1 [file molecules-30-00716-s001.zip › molecules-3405584-supplementary.pdf]

Supplementary Materials

An Eight-Membered Ring Molecular Framework Based on Carbazole for the Development of Electroluminescent Materials

An Yan, Shipan Xu, Xuyang Du, Chenyun Zhu, Shengli Li, Xiaolong Yang*, Guijiang Zhou* and Yuanhui Sun*

School of Chemistry, Xi'an Key Laboratory of Sustainable Energy Material Chemistry, Engineering Research Center of Energy Storage Materials and Devices, Ministry of Education, Xi'an Jiaotong University, Xi'an 710049, China

*Correspondence: xiaolongyang@xjtu.edu.cn (X.Y.); zhougj@xjtu.edu.cn (G.Z.); sunyuanhui@xjtu.edu.cn (Y.S.)

General experimental information

Commercially available starting materials were used directly without further purification. The thermal stability ware tested on a NETZSCH STA 409C instrument under N_2 at a heating rate of 20 K min^{-1}. UV-vis absorption spectra were measured on a Shimadzu UV-2250 spectrophotometer in Toluene at room temperature. Photoluminescent spectra and lifetimes of these compounds were measured on an Edinburgh Instruments Ltd (FLS920) fluorescence spectrophotometer. Absolute photoluminescent quantum yields (PLQYs) of solution samples are obtained using an integrating sphere. ^{1}H NMR and ^{13}C NMR spectra were measured in $CDCl_3$ on a Bruker Avance 400 MHz spectrometer. Chemical shifts were referenced to the solvent residual peak at δ 7.26 ppm for ^{1}H and 77.0 ppm for ^{13}C, respectively. Mass spectra (MS) measurements were performed on WATERS I-Class VION IMS QTof. Calibrated with ferrocene/ferrocenium (Fc/Fc^+) couple, cyclic voltammetry investigations were conducted on a Princeton Applied Research (PARSTAT 2273, Advanced Electrochemical System) equipment in CH_3CN solutions containing *n*-Bu_4NPF_6 (0.1 M) as the supporting electrolyte under a N_2 atmosphere. The scan rate was 100 mV s^{-1}. The HOMO and LUMO energy levels were calculated using the oxidation potential (E_{ox}) and reduction potential (E_{red}) according to the equations $E_{HOMO} = -(E_{ox} + 4.8)$ eV and $E_{LUMO} = -(E_{red} + 4.8)$ eV.

Theoretical calculation

Theoretical calculations were performed for all these eight-membered ring compounds based on density functional theory (DFT) and time dependent-DFT (TD-

DFT) calculations. Non-metal atoms of C, H, N, O, and S were calculated based on B3LYP/6-31G(d, p). The excitation behaviors of the compounds were calculated by TD-DFT method based on optimized geometries at the ground states. All calculations were carried out by using the Gaussian 09 program.

OLED fabrication and measurements.

The relevant devices of luminescent materials were prepared by vacuum evaporation method. The ITO glass substrates were pre-cleaned in ultrasonic cleaner with detergent and deionized water. The substrates were treated with UV-O_3 for 30 minutes before use. Then, HAT-CN (10 nm), TCTA (10 nm), EML (25 nm), TmPyPb (40 nm), LiF 1 (nm), and Al (100 nm) were vacuum deposited under *ca.* 5×10^{-4} torr to complete the devices. The current density-voltage (*I*-*V*) curves were measured by a dual-channel Keithely 2400 Source Meter. The EL spectra and EQEs were measured using and multi-channel analyzer PMA−12. The device performance measurements were carried out under ambient conditions.

Synthesis and Characterization

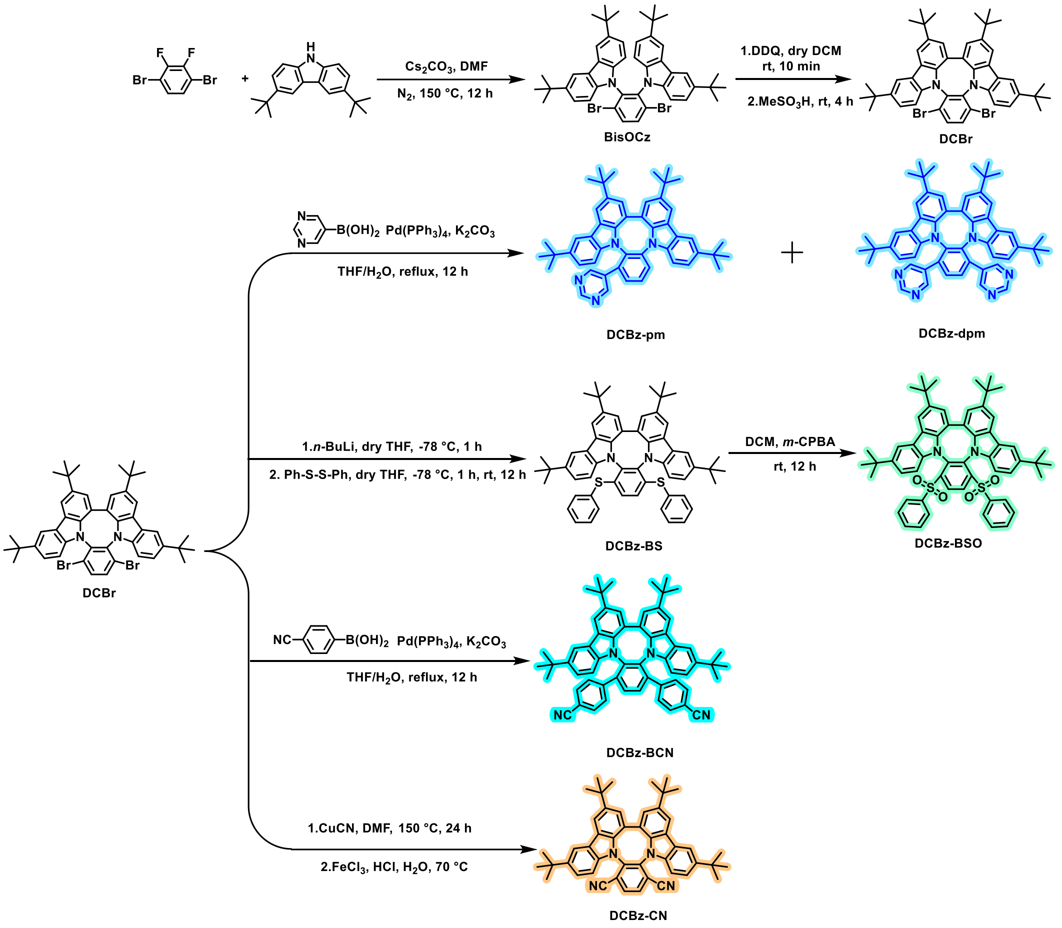

Scheme 1. Synthetic route of the eight-membered ring group

Synthesis of 9, 9'-(3, 6-dibromo-1, 2-phenylene) bis (3, 6-di-tert-butyl-9H-carbazole) (BisOCz): 3, 6-Di-*tert*-butyl carbazole (0.31 g, 1.10 mmol), Cs_2CO_3 (1.01 g, 3 mmol), and ultra-dry DMF (15 mL) were added to a Schlenk reaction tube and stirred for 30 min at room temperature under N_2 atmosphere. Then 1, 4-dibromo-2, 3-difluoro benzene (0.14 g, 0.50 mmol) was added to the mixture, and the mixture was heated and stirred for 12 h at 150 °C under N_2 atmosphere. After the reaction, the mixture was cooled to room temperature, 80 mL of deionized water was added to the mixture, and the crude product yellow filter cake was obtained by suction under reduced pressure. The crude product was dissolved in 20 mL of dichloromethane, and then 50

mL of methanol was added to the solution for recrystallization. The solid-liquid separation was achieved by vacuum filtration again, and the white solid was obtained as the target product BisOCz (0.36 g, 90% yield). ^{1}H NMR (400 MHz, $CDCl_3$) δ 7.88 (s, 2H), 7.52 (d, *J* = 1.60 Hz, 4H), 6.89 (dd, *J* = 8.8, 2.00 Hz, 4H), 6.61 (d, *J* = 8.80 Hz, 4H), 1.30 (s, 36H);

Synthesis of 1, 4-dibromo-8, 11, 14, 17-tetra-*tert*-butylbenzo [2, 3] [1, 4] diazocino [6, 5, 4-jk:7, 8, 1-j'k'] dicarbazole (DCBr): BisOCz (0.79 g, 1.00 mmol) and 2, 3-dichloro-5, 6-dicyano-1, 4-benzoquinone (DDQ) (0.34 g, 1.50 mmol), dried dichloromethane (50 mL) was added to a 250 ml Schlenk reaction tube. The mixture was stirred for 10 min at room temperature under N_2 atmosphere. Then 0.50 mL (0.76 mmol) of mesylate was added to the mixture and the stirring continued for 4 h at room temperature before quenching by adding 10 mL triethylamine. The solvent was removed by distillation under reduced pressure, and the crude product was purified by silica gel column chromatography with hexane: dichloromethane (10:1, *v/v*) as eluent to obtain a white solid, DCBr (0.50 g, 66% yield). ^{1}H NMR (400 MHz, $CDCl_3$) δ 7.99 (s, 4H), 7.85 (s, 2H), 7.45 (dd, *J* = 8.4, 1.60 Hz, 2H), 7.30 (s, 2H), 7.14 (d, *J* = 8.80 Hz, 2H), 1.47 (s, 18H), 1.43 (s, 18H);

Synthesis of 8, 11, 14, 17-tetra-*tert*-butyl-1- (pyrimidin-5-yl) benzo [2, 3] [1, 4] diazocino [6, 5, 4-jk:7, 8, 1-j'k'] dicarbazole (DCBz-pm): DCBr (0.11 g, 0.13 mmol), 5-pyrimidine boric acid (36 mg, 0.30 mmol), and Pd $(PPh_3)_4$ (15 mg, 0.01 mmol) were added to a 100 ml Schlenk reaction tube after N_2 was drained three times, tetrahydrofuran (15 mL) and K_2CO_3 aqueous solution (10 mL, 2 M) were added to the

reaction tube, and then heated and stirred under reflux for 12 h in a N_2 atmosphere. 5-pyrimidine boric acid was added during the reaction. After the reaction, the mixture was cooled to room temperature, extracted three times with dichloromethane (15 mL×3), merged the organic phase and dried with anhydrous Na_2SO_4, filtered through a sand-core funnel to remove the catalyst residue, and distilled again under reduced pressure to remove the solvent to give a crude yellow solid. The product was purified by thin layer chromatography with dichloromethane: ethyl acetate (20:1, *v/v*) as the development agent to obtain a white solid, DCBz-pm (38 mg, 43% yield). ^{1}H NMR (400 MHz, $CDCl_3$) δ 8.69 (s, 1H), 8.10 (s, 1H), 8.08 (s, 2H), 7.99 (s, 2H), 7.87 (d, J = 4.80 Hz, 2H), 7.56 (s, 1 H), 7.43 (d, J = 8.40 Hz, 1 H), 7.32 (d, J = 8.40 Hz, 1H), 7.24, 7.18 (m, 3H), 6.77, 6.74 (m, 2H), 1.53 (s, 9H), 1.47 (s, 9H), 1.45 (s, 9H), 1.33 (s, 9H); ^{13}C NMR (100 MHz, $CDCl_3$) δ157.05, 154.96, 147.89, 147.46, 147.01, 146.66, 146.61, 143.74, 143.39, 139.63, 139.19, 135.44, 133.35, 131.79, 130.60, 130.33, 129.73, 128.35, 128.21, 127.45, 125.80, 125.22, 124.72, 124.12, 123.60, 121.98, 116.98, 116.77, 116.51, 116.29, 115.94, 111.52, 35.04 (2C), 34.80, 34.68, 31.99, 31.93 (2C); MS(m/z): 709.4296[M+H]$^+$. Calculated For $C_{50}H_{52}N_4$: 708.4192.

Synthesis of 8, 11, 14, 17-tetra-*tert*-butyl-1, 4-di (pyrimidin-5-yl) benzo [2, 3] [1, 4] diazocino [6, 5, 4-jk:7, 8, 1-j'k'] dicarbazole (DCBz-dpm): The target product DCBz-dpm (42 mg, 42% yield) was obtained by the same synthesis method as DCBz-pm. ^{1}H NMR (400 MHz, $CDCl_3$) δ 8.73 (s, 2H), 8.12 (s, 2H), 7.89 (s, 2H), 7.70 (s, 4H), 7.66 (s, 2H), 7.23 7.19 (m, 4H), 6.95 (d, J = 8.80 Hz, 2H), 1.54 (s, 18H), 1.32 (s, 18H); ^{13}C NMR (100 MHz, $CDCl_3$) δ157.19, 154.81, 145.82, 144.96, 142.42, 139.22, 135.53,

132.19, 130.70, 128.19, 126.82, 125.81, 124.21, 123.69, 116.93, 116.41, 113.57, 35.05, 34.74, 31.99, 31.76; MS(m/z): 787.4475$[M+H]^+$. Calculated For $C_{54}H_{54}N_6$: 786.4405.

Synthesis of 8, 11, 14, 17-tetra-*tert*-butyl-1, 4-bis (phenylthio) benzo[2, 3] [1, 4] diazocino [6, 5, 4-jk:7, 8, 1-j'k'] dicarbazole (DCBz-BS): DCBr (0.30 g, 0.38 mmol) and ultra-dry THF (10 mL) were added to a 100 ml Schlenk reaction tube. After three times of N_2 extraction, the temperature of the reaction system was reduced to –78 °C by an ethyl acetate/liquid nitrogen bath n-BuLi hexane solution (0.40 mL, 2.50 M) was added to it drop by drop, and the temperature was gradually raised to –40 °C and maintained for 1 h. Then the reaction system was cooled to –78 °C again, and THF (10 mL) solution of diphenyl disulfide (0.18 g, 0.84 mmol) was added to it drop by drop. After the completion of the drop, the mixture was maintained at –78 °C for 1 h and gradually returned to room temperature for 12 h with stirring. At the end of the reaction, 10 mL deionized water was added to quench the excess n-BuLi, dichloromethane (12 mL×3) was used for extraction three times, the organic phase was combined and dried with anhydrous Na_2SO_4, and the solvent was removed by distillation under reduced pressure to obtain the crude yellow solid. The target product DCBz-BS was purified by silica gel column chromatography with petroleum ether dichloromethane (5:1, *v/v*) as eluent, and a white solid (0.22 g, 69% yield) was obtained. ^1H NMR (400 MHz, $CDCl_3$) δ8.05 (d, J = 1.60 Hz, 2H), 7.99 (d, J = 2.00 Hz, 2H), 7.84 (d, J = 1.60 Hz, 2H), 7.45 (dd, 2H) J = 8.4, 1.60 Hz, 2H), 7.21 (d, J = 8.40 Hz, 2H), 7.09 7.03 (m, 6H), 6.86 6.83 (m, 4H), 6.77 (s, 2H), 1.48 (s, 18H), 1.43 (s, 18H);

Synthesis of 8, 11, 14, 17-tetra-*tert*-butyl-1, 4-bis (phenylsulfonyl) benzo [2, 3] [1,

4] diazocino [6, 5, 4-jk:7, 8, 1-j'k'] dicarbazole (DCBz-BSO): DCBz-BS (0.10 g, 0.12 mmol) and dichloromethane (20 mL) were added to a 100 mL round-bottom flask, m-chlorperoxybenzoic acid (62 mg, 0.36 mmol) was added slowly, and the mixture was stirred for 12 h at room temperature. At the end of the reaction, the solvent was removed by distillation under reduced pressure to give the crude product a yellow solid. The crude product was purified by silica gel TLC using petroleum ether: THF (2:1, *v/v*) as the development agent, and the yellow solid was obtained as the target product DCBz-BSO (0.09 g, 82% yield). ^{1}H NMR (400 MHz, $CDCl_3$) δ 8.15 (s, 2H), 7.98 (s, 2H), 7.70 (s, 2H), 7.58 (d, J = 8.40 Hz, 2H), 7.55 (s, 2H), 7.40 (d, J = 8.40 Hz, 2H) J = 8.4 Hz, 2H), 7.15 (t, J = 7.20 Hz, 2H), 6.96 (t, J = 7.60 Hz, 4H), 6.38 (d, J = 7.60 Hz, 4H), 1.48 (s, 18H), 1.45 (s, 18H); ^{13}C NMR (100 MHz, $CDCl_3$) δ144.58, 144.45, 143.31, 140.85, 139.48, 136.01, 134.96, 132.95, 131.56, 128.89 (2C), 127.61, 126.53, 124.92, 123.99 (2C), 116.35, 115.52 112.87, 34.85, 34.81, 31.99, 31.92; MS(m/z): 911.3866$[M+H]^+$. Calculated For $C_{58}H_{58}N_2O_4S_2$: 910.3838.

Synthesis of 4, 4'-(8, 11, 14, 17-tetra-*tert*-butylbenzo [2, 3] [1, 4] diazocino [6, 5, 4-jk:7, 8, 1-j'k'] dicarbazole-1, 4-diyl) dibenzonitrile (DCBz-BCN): DCBr (100 mg, 0.13 mmol), 4-cyanophenylboronic acid (43 mg, 0.30 mmol), and Pd $(PPh_3)_4$ (15 mg, 0.01 mmol) were added to a 100 ml Schlenk reaction tube, and N_2 was drained three times. Tetrahydrofuran (15 mL) and K_2CO_3 aqueous solution (10 mL, 2 M) were added to the reaction tube, followed by heating, and stirring under reflux for 12 h in a N_2 atmosphere. After the reaction, the mixture was cooled to room temperature, extracted three times with dichloromethane (13 mL×3), merged the organic phase and dried with

anhydrous Na_2SO_4, filtered through a sand-core funnel to remove the catalyst residue, and distilled again under reduced pressure to remove the solvent to give a crude yellow solid. The target product DCBz-BCN (0.07 g, 69% yield) was purified by silica gel TLC with petroleum ether dichloromethane (3:2, *v/v*) as a development agent. ^1H NMR (400 MHz, $CDCl_3$) δ 8.15 (s, 2H), 7.80 (s, 2H), 7.54 (s, 2H), 7.19 (s, 2H), 7.11 (d, J = 8.00 Hz, 2H), 7.03 (d, j = 8.00 Hz, 2H) J = 7.6 Hz, 4H), 6.92 (d, J = 8.80 Hz, 2H), 6.41 (s, 4H), 1.55 (s, 18H), 1.31 (s, 18H); ^{13}C NMR (100 MHz, $CDCl_3$) δ145.25, 144.54, 142.67 (2C), 142.47, 139.87, 138.46, 130.70, 129.72, 128.54, 128.44, 126.51, 124.77, 123.59, 123.31, 118.65, 116.51, 115.64, 113.62, 110.48, 34.95, 34.60, 31.98, 31.79; MS(m/z): 833.4548$[M+H]^+$. Calculated For $C_{60}H_{56}N_4$: 832.4505.

Synthesis of 8, 11, 14, 17-tetra-*tert*-butylbenzo [2, 3] [1, 4] diazocino [6, 5, 4-jk:7, 8, 1-j'k'] dicarbazole-1, 4-dicarbonitrile (DCBz-CN): DCBr (0.10 g, 0.13 mmol) and ultra-dry DMF (20 mL) were added to a 100 mL Schlenk reaction tube and heated to 140 °C under N_2 atmosphere. After the solution became clear, CuCN (68 mg, 0.76 mmol) was added and continued to stir for 24 hours. Weigh 1.50 g ferric chloride solid to prepare a saturated aqueous solution, add concentrated hydrochloric acid (1.50 mL, 12 M) to it, then slowly add the mixed solution to the cooled reaction solution, and heat and stir at 70 °C for 30 min. After cooling the reaction liquid, 20 mL of deionized water was added and extracted three times with ethyl acetate (19 mL×3), the organic phase was combined and dried with anhydrous Na_2SO_4, and the solvent was removed by distillation under reduced pressure to give an orange solid crude product. The target product DCBz-CN (0.07 g, 69% yield) was purified by silica gel TLC with petroleum

ether: ethyl acetate (5:1, *v/v*) as a development agent. ^{1}H NMR (400 MHz, $CDCl_3$) δ 8.07 (s, 2H), 7.96 (s, 2H), 7.87 (s, 2H), 7.54 (s, 2H), 7.52 (d, J = 8.80 Hz, 2H), 7.23 (d, J = 8.80 Hz, 2H) J = 8.80 Hz, 2H), 1.48 (s, 18H), 1.47 (s, 18H); ^{13}C NMR (100 MHz, $CDCl_3$) δ147.11, 146.50, 143.89, 143.24, 141.06, 133.31, 129.07, 128.38, 126.58, 124.79, 123.86, 117.00, 116.90, 113.70, 113.29, 35.06, 35.00, 31.94 (2C); MS(m/z): 681.3945$[M+H]^+$. Calculated For $C_{48}H_{48}N_4$: 680.3879.

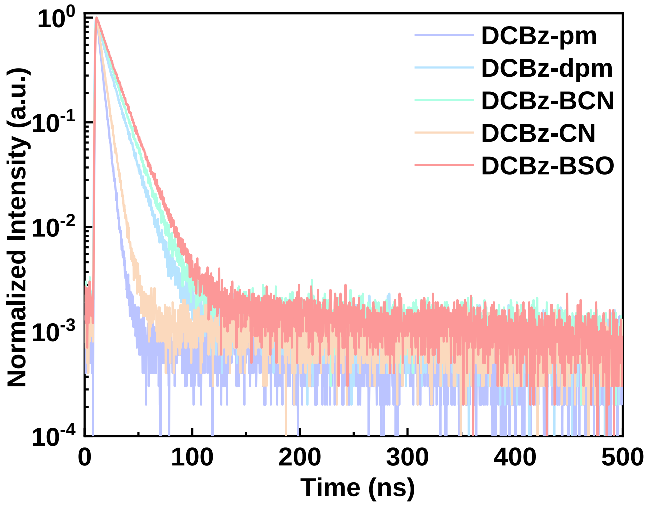

Figure S1. The transient photoluminescence decay curves under degassed conditions in toluene solution at room temperature.

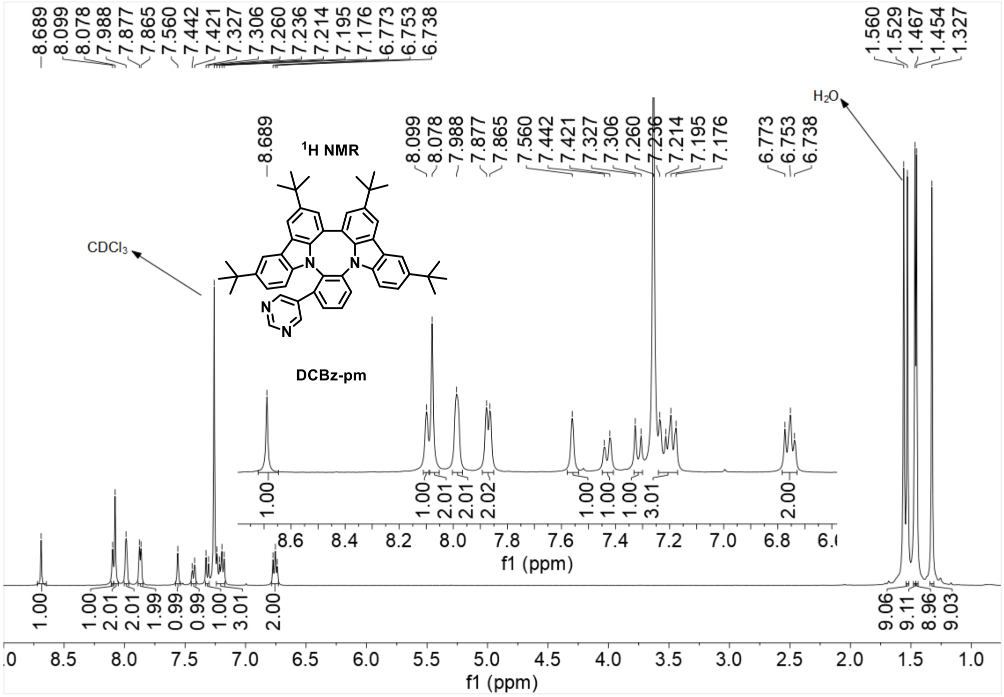

Figure S2. ^{1}H NMR spectra of DCBz-pm.

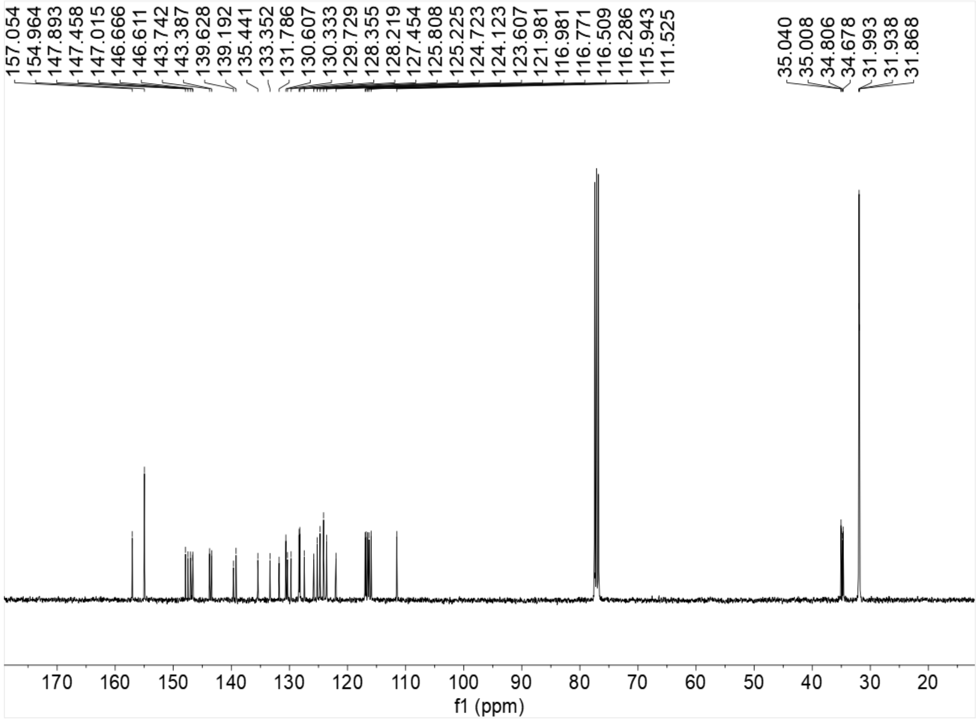

Figure S3. ^{13}C NMR spectra of DCBz-pm.

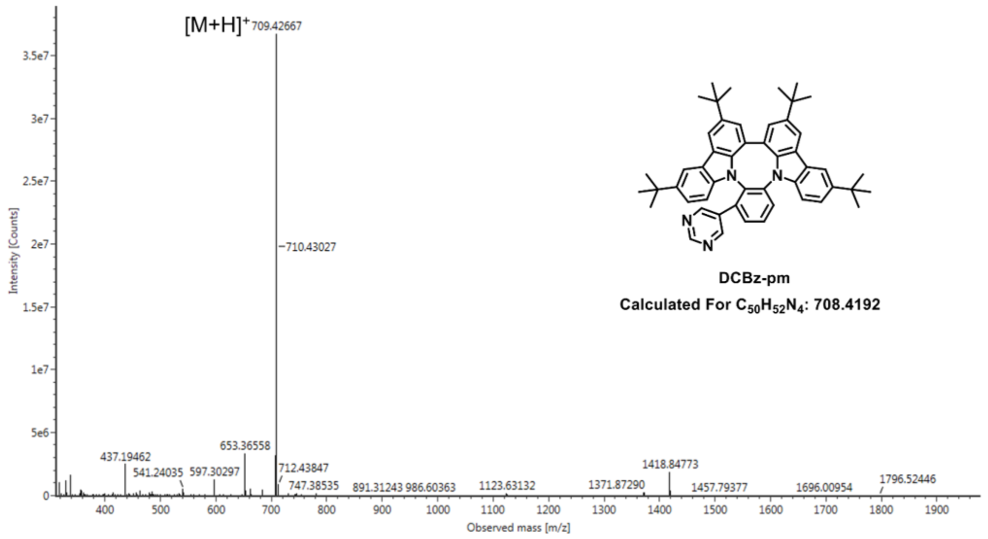

Figure S4. MS spectra of DCBz-pm.

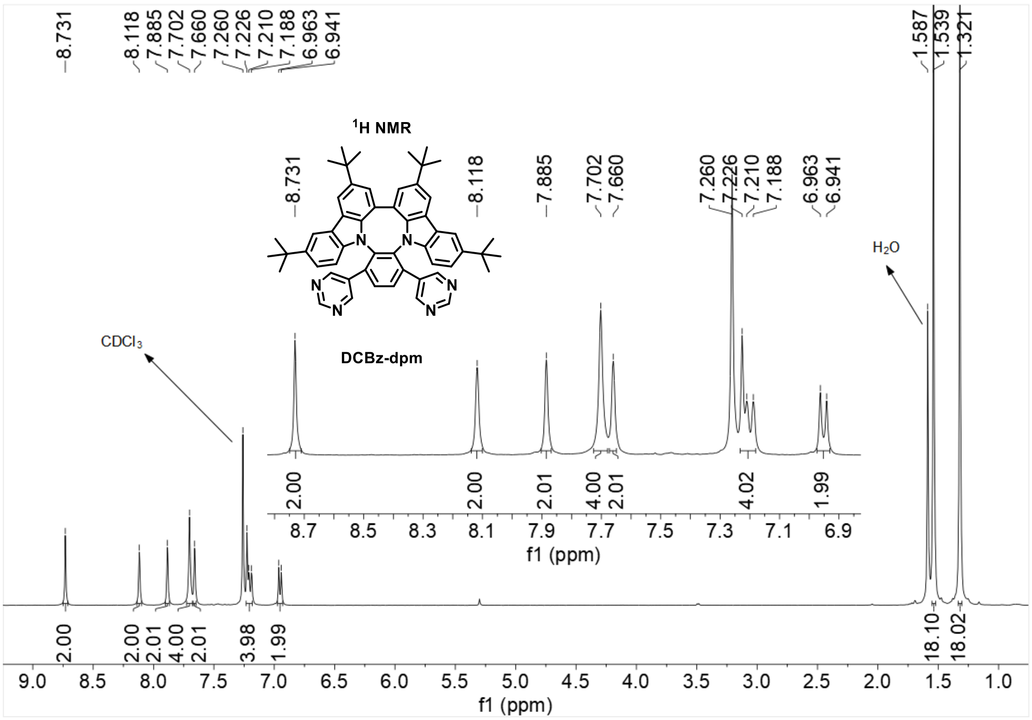

Figure S5. ^{1}H NMR spectra of DCBz-dpm.

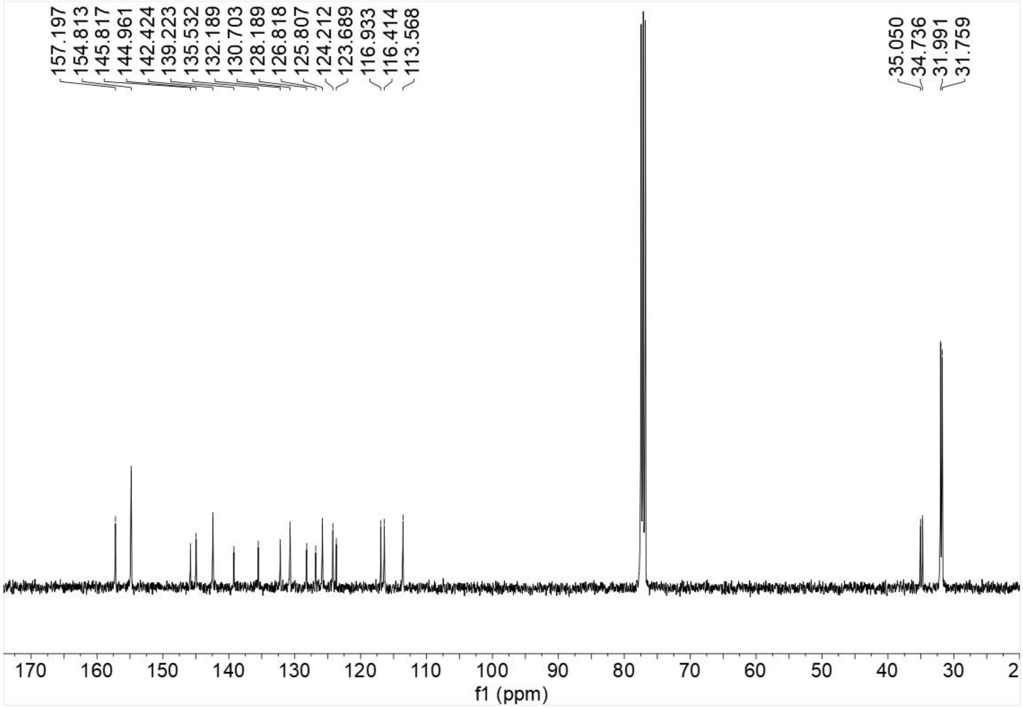

Figure S6. ^{13}C NMR spectra of DCBz-dpm.

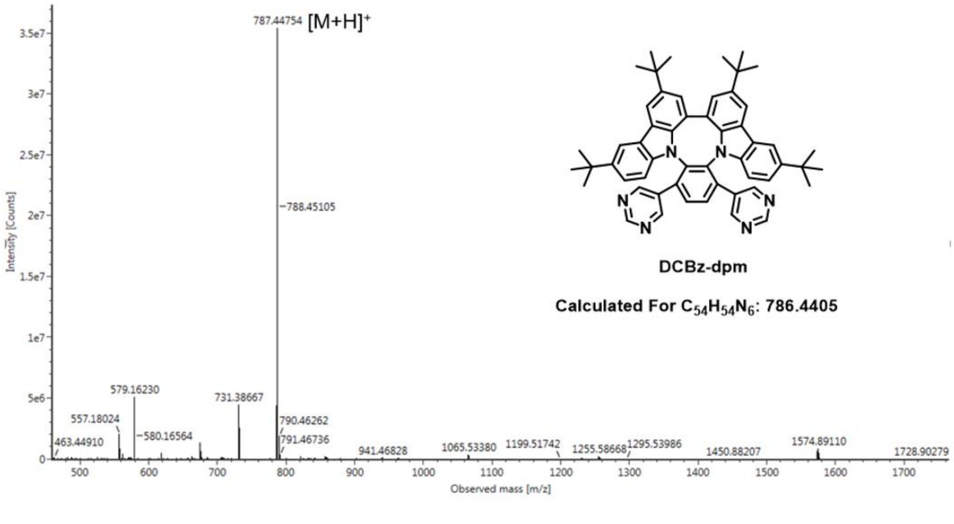

Figure S7. MS spectra of DCBz-dpm.

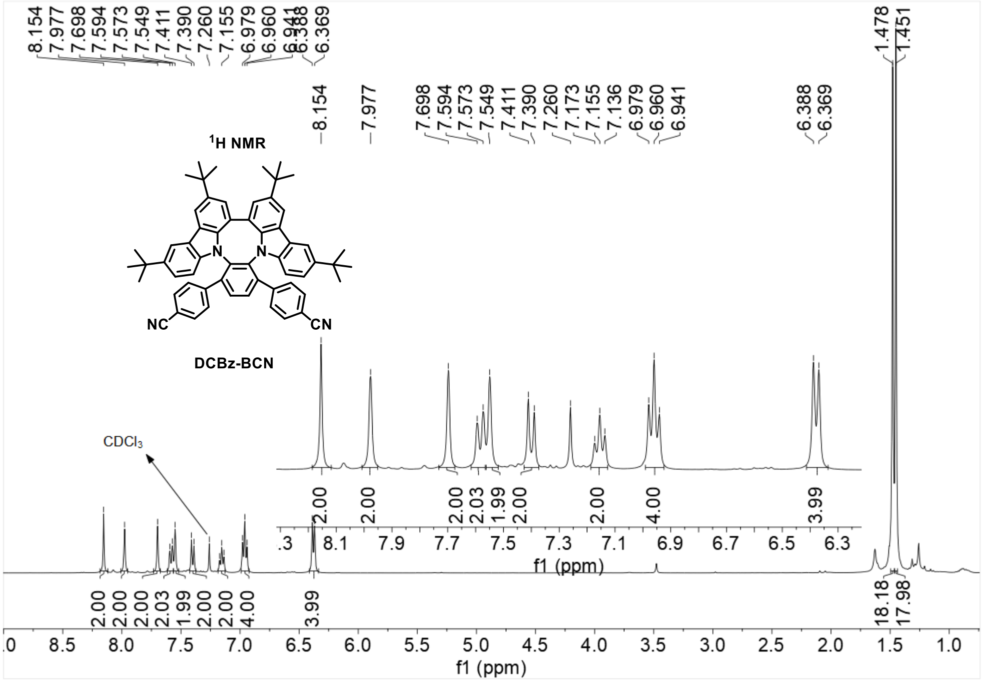

Figure S8. ^{1}H NMR spectra of DCBz-BCN.

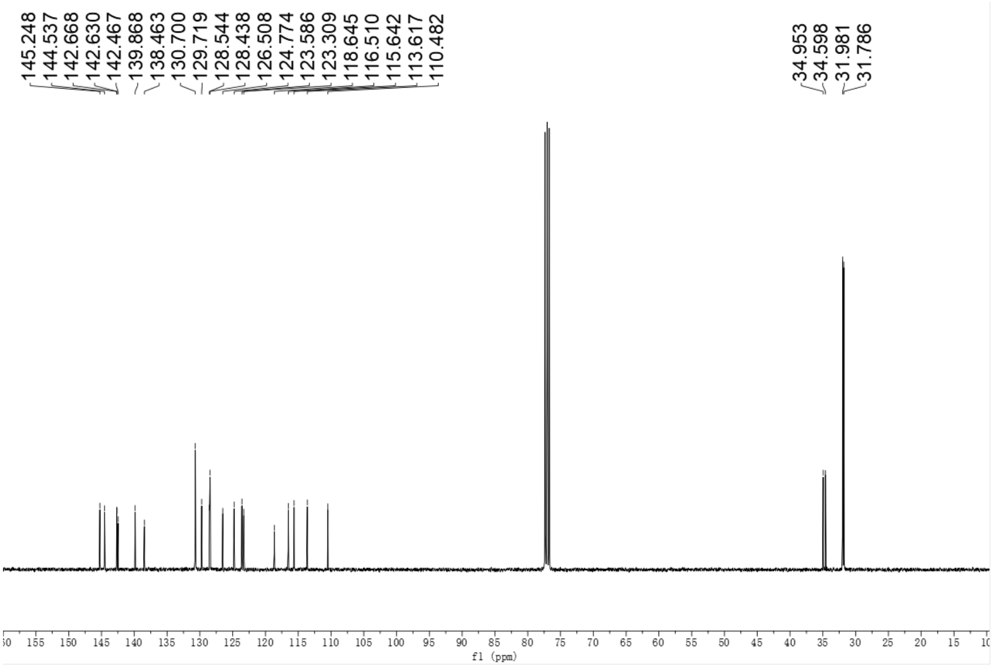

Figure S9. ^{13}C NMR spectra of DCBz-BCN.

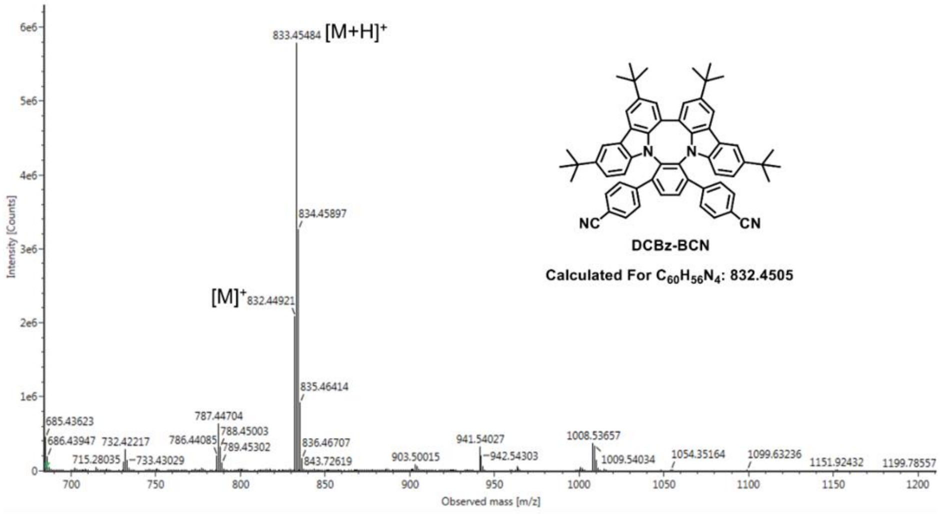

Figure S10. MS spectra of DCBz-BCN.

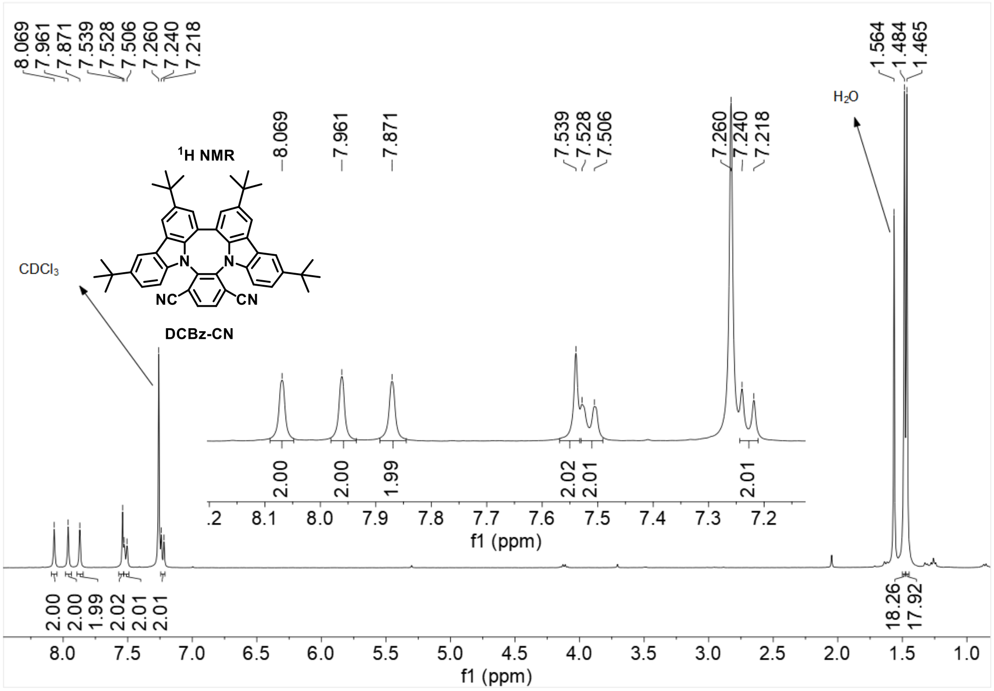

Figure S11. ^{1}H NMR spectra of DCBz-CN.

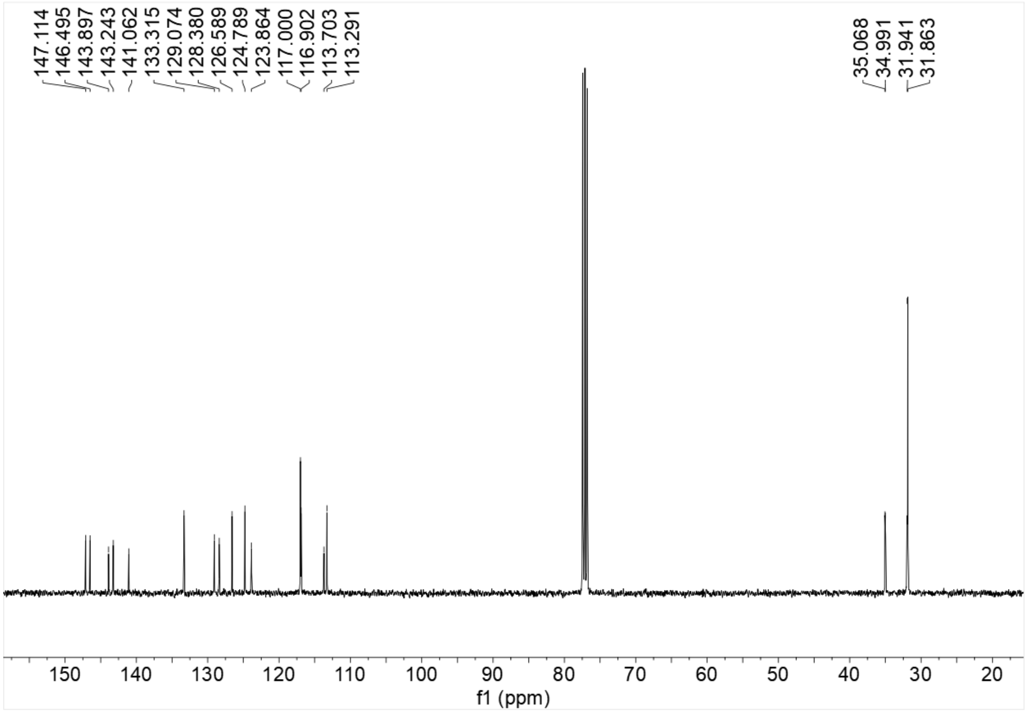

Figure S12. ^{13}C NMR spectra of DCBz-CN.

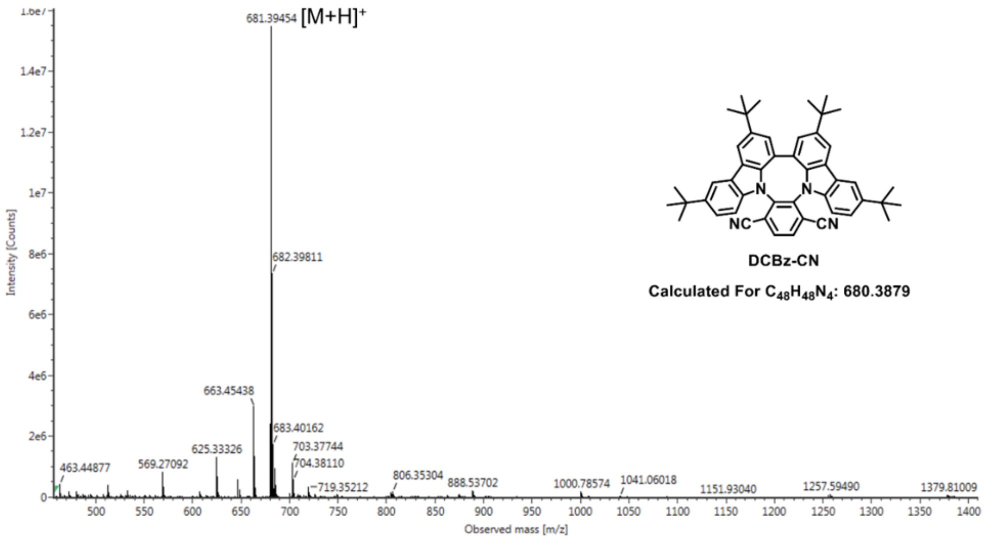

Figure S13. MS spectra of DCBz-CN.

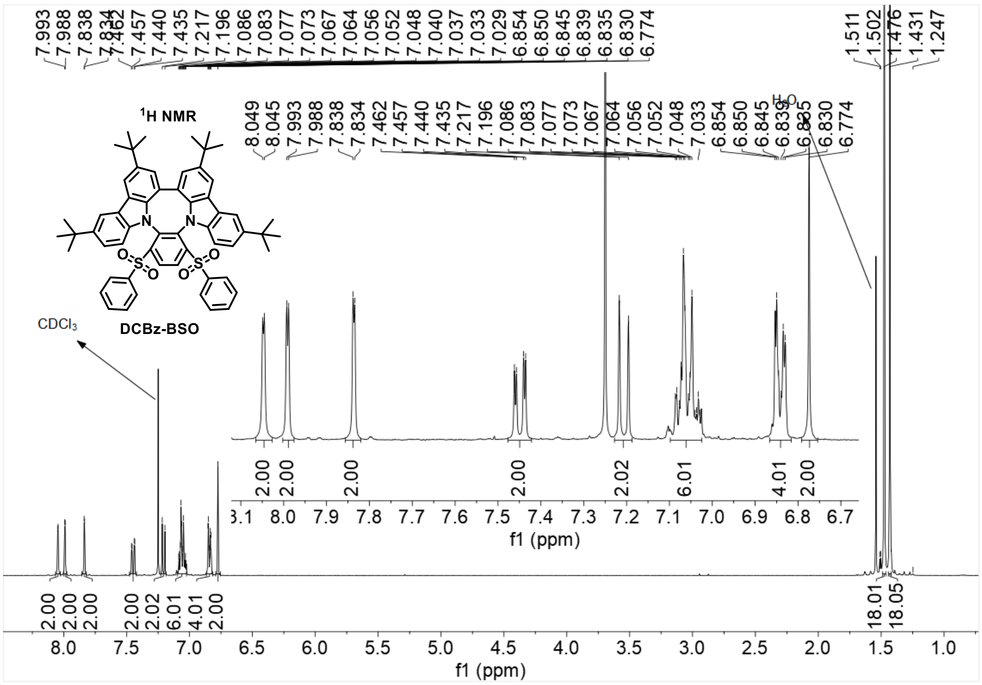

Figure S14. ^{1}H NMR spectra of DCBz-BSO.

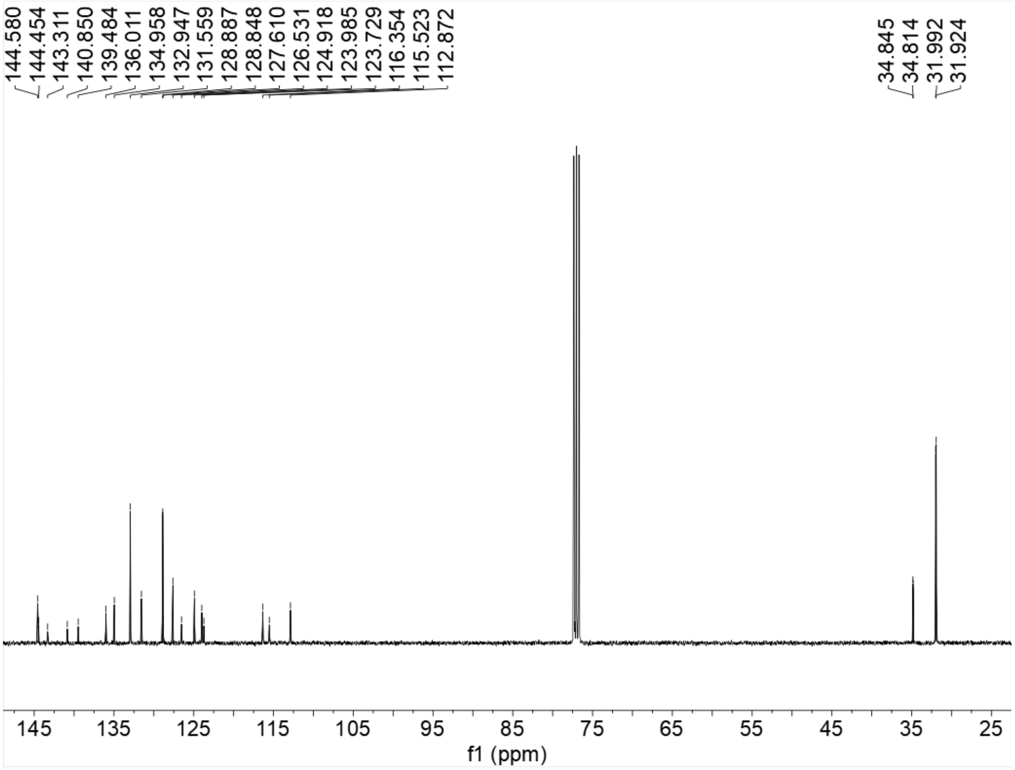

Figure S15. ^{13}C NMR spectra of DCBz-BSO.

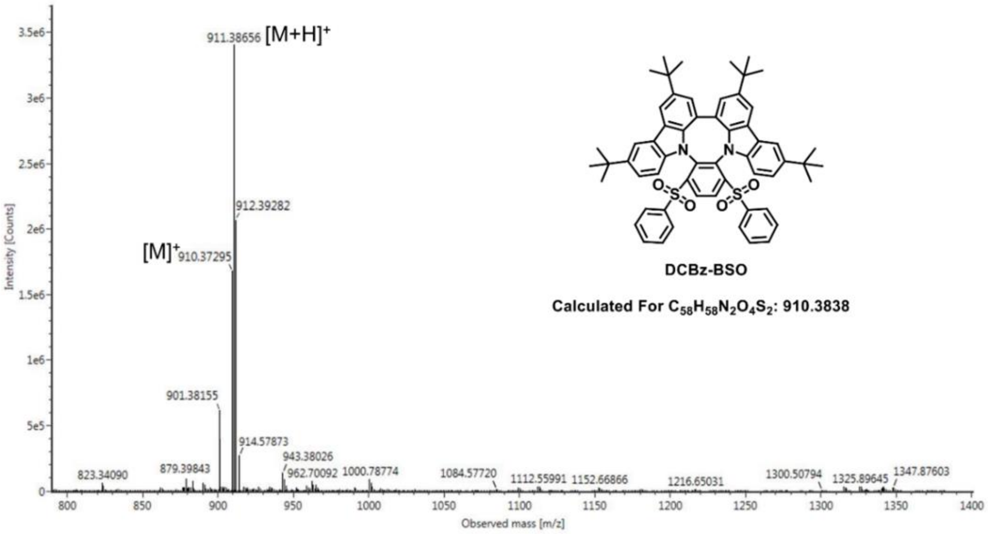

Figure S16. MS spectra of DCBz-BSO.
